# Infant Brain Structural MRI Analysis in the Context of Thoracic Non-cardiac Surgery and Critical Care

**DOI:** 10.3389/fped.2019.00315

**Published:** 2019-08-02

**Authors:** Chandler R. L. Mongerson, Sophie L. Wilcox, Stacy M. Goins, Danielle B. Pier, David Zurakowski, Russell W. Jennings, Dusica Bajic

**Affiliations:** ^1^Department of Anesthesiology, Critical Care and Pain Medicine, Boston Children's Hospital, Boston, MA, United States; ^2^Massachusetts General Hospital Child Neurology, Boston, MA, United States; ^3^Harvard Medical School, Harvard University, Boston, MA, United States; ^4^Department of Surgery, Boston Children's Hospital, Boston, MA, United States

**Keywords:** brain atrophy, cerebrospinal fluid, full-term, long-gap esophageal atresia, prematurity, sedation, MANTiS, volumetric analysis

## Abstract

**Objective:** To determine brain magnetic resonance imaging (MRI) measures of cerebrospinal fluid (CSF) and whole brain volume of full-term and premature infants following surgical treatment for thoracic non-cardiac congenital anomalies requiring critical care.

**Methods:** Full-term (*n* = 13) and pre-term (*n* = 13) patients with long-gap esophageal atresia, and full-term naïve controls (*n* = 19) < 1 year corrected age, underwent non-sedated brain MRI following completion of thoracic non-cardiac surgery and critical care treatment. Qualitative MRI findings were reviewed and reported by a pediatric neuroradiologist and neurologist. Several linear brain metrics were measured using structural T1-weighted images, while T2-weighted images were required for segmentation of total CSF and whole brain tissue using the **M**orphologically **A**daptive **N**eonatal **T**issue **S**egmentation (**MANTiS**) tool. Group differences in absolute (mm, cm^3^) and normalized (%) data were analyzed using a univariate general linear model with age at scan as a covariate. Mean normalized values were assessed using one-way ANOVA.

**Results:** Qualitative brain findings suggest brain atrophy in both full-term and pre-term patients. Both linear and volumetric MRI analyses confirmed significantly greater total CSF and extra-axial space, and decreased whole brain size in both full-term and pre-term patients compared to naïve controls. Although linear analysis suggests greater ventricular volumes in all patients, volumetric analysis showed that normalized ventricular volumes were higher only in premature patients compared to controls.

**Discussion:** Linear brain metrics paralleled volumetric MRI analysis of total CSF and extra-axial space, but not ventricular size. Full-term infants appear to demonstrate similar brain vulnerability in the context of life-saving thoracic non-cardiac surgery requiring critical care as premature infants.

## Introduction

It is known that prematurity [defined as birth <37 weeks gestational age (GA)], is associated with a plethora of negative neurologic long-term sequelae, as previously shown in studies spanning neurobehavioral (1–3), cognitive (4–6), and neuroimaging domains (7). Indeed, emerging evidence for developmental plasticity suggests early exposure to stressors during neonatal intensive care, such as procedural pain or prolonged exposure to analgesic medications, may disrupt normal processes of brain maturation (8–11). Furthermore, recent studies reported that altered regional brain development was associated with critical illness (e.g., chronic lung disease, necrotizing enterocolitis, patent ductus arteriosus, postnatal infection, and need for mechanical ventilation) irrespective of prematurity (12, 13).

Little is known regarding the impact of complex perioperative care for non-cardiac congenital anomalies on neurodevelopmental outcomes in full-term infants. As survival rates continue to improve for critically-ill infants born with non-cardiac congenital anomalies (14, 15), concerns have been raised regarding incidence of brain injury (16) and long-term neurodevelopmental delay (17, 18) following surgery and complex critical care. Although the etiology is unknown, studies in full-term infants with congenital diaphragmatic hernia have implicated extracorporeal membrane oxygenation (ECMO) as a potential risk factor for adverse neurodevelopmental outcomes (19, 20). Currently, little is known regarding incidence of brain abnormalities following neonatal surgical and critical care treatment for thoracic non-cardiac congenital anomalies in the absence of ECMO (16). At our institution, the premier Esophageal and Airway Treatment Center offers the opportunity to study a unique population of full-term and premature infants born with thoracic non-cardiac gastrointestinal congenital anomalies [viz. long-gap esophageal atresia (LGEA) (21, 22)] that require surgical treatment without the confounds of ECMO. The aim of this study was to evaluate qualitative and quantitative measures of brain and cerebrospinal fluid (CSF) using magnetic resonance imaging (MRI) in full-term and moderate-to-late pre-mature (born between 28 and 36 weeks GA) infants undergoing life-saving surgery for non-cardiac LGEA requiring critical care (in the absence of any known neurological problems). Quantitative measures included simple linear metrics (23–26) and volumetric analysis (27). We hypothesized that both full-term and premature critically-ill patients <1 year-old, compared to healthy infants (as normative controls), would exhibit (1) higher incidence of brain findings consistent with brain atrophy, (2) greater CSF, and (3) smaller brain tissue measures following complex perioperative critical care.

## Methods

### Study Design and Participants

**Figure 1:**
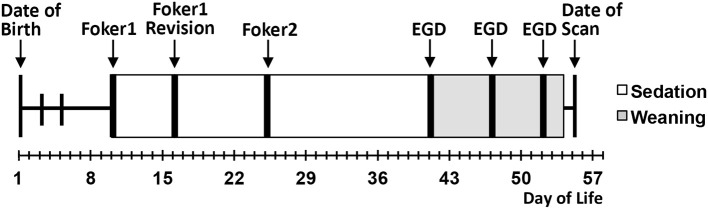
Schematic illustrates a single representative case of thoracic non-cardiac surgical [Foker process (21, 22)] and critical care treatment timeline for long-gap esophageal atresia (LGEA) repair that includes prolonged sedation to maintain mechanical ventilation (white boxes), as well as subsequent weaning of sedation drugs (gray boxes). Research brain MRI scan was done after completion of all treatment. EGD, esophagoduodenoscopy.

We conducted a pilot infant MRI study with ethical approval from Boston Children's Hospital Review Board as a “no more than minimal risk” study. Informed written consent was obtained from parents before participation, in accordance with the Declaration of Helsinki and Good Clinical Practice. Patient eligibility criteria included <1-year-old born *full-term* (37–42-weeks GA) or moderate-to-late *pre-term* (28–36-weeks GA) infants who underwent thoracic non-cardiac surgery for gastrointestinal congenital anomaly [viz. Foker process for LGEA repair (21, 22)] that required complex perioperative critical care: (1) prolonged sedation [>5 days, associated with development of drug dependence (28, 29)], and (2) subsequent weaning from sedation medications. Illustrative timeline of perioperative critical care is summarized in Figure 1. Healthy full-term naïve infants were recruited as a comparative baseline for typical infant brain development. Exclusion criteria included: (1) cardiac surgeries and/or ECMO exposure; (2) MRI incompatible implants; (3) cranial ultrasound findings (e.g., ventricular enlargement, hemorrhage); (4) chromosomal abnormalities (e.g., Down Syndrome); (5) neurological disease (e.g., seizures); (6) prenatal drug exposure; and/or (7) extreme prematurity (<28 weeks GA). Subjects were categorized into 3 groups: full-term patients, premature patients, or full-term naïve controls. Table 1 displays a summary of the number of subjects screened, excluded, and enrolled by group.

**Table 1:** Recruitment and scanning summary.

***n***	**Naïve controls**	**Full-term patients**	**Pre-term patients**
Considered/(chart) reviewed	59	173	107
**Eligible** (% reviewed)	**56** (95%)	**63** (36%)	**49** (46%)
Approached (% eligible)	53 (95%)	40 (63%)	23 (47%)
Consented (% approached)	19 (36%)	19 (48%)	18 (78%)
Scanned (% consented)	19 (100%)	13 (68%)	13 (72%)
**Included/Analyzed** (% scanned)	**19** (100%)	**13** (100%)	**13** (100%)

*Table summarizes infant MRI study recruitment process by group (naïve controls, full-term and pre-term patients). Major reasons for non-scanning of consented patients were related to either infants' health or scheduling factors. All successfully scanned subjects were included in the analysis (100% scanned). Bolded text highlights the difference in eligible number of subjects and those that underwent successful scans. Table 2 shows further analysis details*.

### MRI Acquisition

All infants underwent non-sedated research scan in a 3T TrioTim MRI system equipped with 32-channel receive-only head coil and body-transmission (Siemens Healthcare Inc., USA) following completion of all perioperative care.

#### Preparation for Non-sedated MRI

To improve infant's compliance during neuroimaging sessions in the research setting, we followed previously described practices of natural sleep technique and the “feed and wrap” approach (30–33). All imaging was done during infants' sleeping time (evenings and night) and parents were encouraged to perform sleep routine (e.g., nursing, rocking, singing, etc.). Infants were bundled and cradled in MRI-safe Deluxe+ carrycot (DockATot, Wilmington, NC), which allowed for easier moving and positioning of sleeping baby into the scanner. Once on the scanner table, smaller infants were swaddled into the beanbags (viz. gentle vacuum bag immobilizer), while infants >3 months were allowed to assume more relaxed position (e.g., arms next to face) before being covered and belted. Both foam earplugs (Newmatic Medical, Birmingham, AL) and earmuffs (MRI-Safe Neonatal Noise Guards, Universal Medical, Norwood, MA) were placed for noise protection while sides of the head were padded with sponges and soft sheets. All infants were continuously monitored for stable heart rate and oxygenation, and one of the parents/guardians was always allowed to stay with the baby in the imaging suite throughout MRI acquisition. Our imaging success rate for healthy infants (76%; Table 1) was similar to that previously reported in literature [see Review (32)]. Our 100% scanning success rate with both patient groups could be attributed to the fact that patients were more acclimated to hospital environment (e.g., more noise; frequent sleep interruptions, etc.) and the fact that we were able to reserve a longer evening/night MRI slot allowing more time for infants to fall asleep.

#### MRI Sequences

T1- and T2-weighted images were acquired using MEMPRAGE sequence [TR/TE = 2,520/1.74 ms; FA = 7°; FOV = 192 × 192 mm^2^; voxels = 1 × 1 × 1 mm^3^] and FSE sequence [TR/TE = 12,624/110 ms; FA = 120°; FOV = 180 × 180 mm^2^; 63 slices, 2 mm thickness; voxels = 0.35 × 0.35 mm^2^], respectively. T1 images were successfully collected for all patients (*n* = 13/group) and 17/19 controls. T2 images were successfully collected for all patients (*n* = 13/group) and 13/19 controls. Clinical characteristics of subjects included for each analysis are summarized in Table 2.

**Table 2:** Clinical characteristics of research subjects.

**Characteristics**	**Naïve controls (*****n*** **= 19)**		**Full-term patients**	**Pre-term patients**
	**T1-weighted (*n* = 17)**	**T2-weighted (*n* = 13)**	**Both scans (*n* = 13)**	**Both scans (*n* = 13)**
Sex (male), *n* (%)	15 (88%)	12 (92%)	7 (54%)	8 (62%)
Average GA at birth (weeks) (SD)	39.5 (0.9)	39.6 (1.0)	38.5 (1.1)	32.2 (2.9)
Median CA at scan [range] (months)	3.2 [0.4–12.3]	2.8 [0.4–7.1]	4.7 [0.6–13.0]	3.8 [1.4–7.5]
Multiple births, *n* (%)	1 (6%)	1 (8%)	1 (8%)	2 (15%)
**INCIDENCE OF MAJOR DIAGNOSES:**				
Isolated LGEA, *n* (%)	0	0	3 (23%)	3 (23%)
LGEA with TEF, *n* (%)	0	0	5 (38%)	9 (69%)
Other, *n* (%)	0	0	5 (38%)	1 (8%)

*This table summarizes clinical characteristics of all subjects included in the quantitative (T1- and T2-weighted) analyses. Patients were stratified into clinical groups of non-cardiac congenital anomalies: (1) isolated LGEA, (2) LGEA with TEF, and (3) other (included LGEA as part of VACTERL association (without cardiac involvement) and/or necrotizing enterocolitis). GA, gestational age; CA, corrected age; LGEA, long-gap esophageal atresia; TEF, tracheo-esophageal fistula; VACTERL stands for vertebral defects, anal atresia, cardiac defects, tracheo-esophageal fistula, renal anomalies, and limb abnormalities*.

### Qualitative MRI

Both T1 and T2-weighted MRI scans (*n* = 13/per patient group; *n* = 19 naïve controls; Table 1) were independently reviewed for incidental findings by the pediatric neuroradiologist on call and a neonatal neurologist (D. Pier). Findings were divided into three categories based on likelihood of brain atrophy as follows: (1) no atrophy (no incidental findings), (2) possible atrophy (1–2 isolated findings), or (3) very likely atrophy (≥3 findings; e.g., concomitant ventriculomegaly, widened Sylvian fissures, and increased extra-axial space and/or interhemispheric fissure). The latter is in accordance with the definition of *cerebral atrophy*: compensatory enlargement of CSF spaces due to reduced brain parenchymal volume.

### Linear Brain Metrics

**Figure 2:**
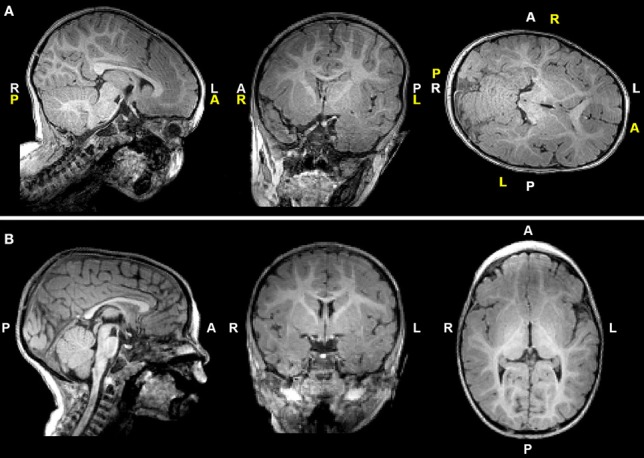
T1-weighted images of a representative full-term infant before **(A)** and after **(B)** manual realignment using Freeview (see Methods). Header information (white letters) is inaccurate in **(A)** due to rotated and tilted head position; corrected labels are shown in yellow letters for reference. A, anterior; L, left; P, posterior; R, right.

**Figure 3:**
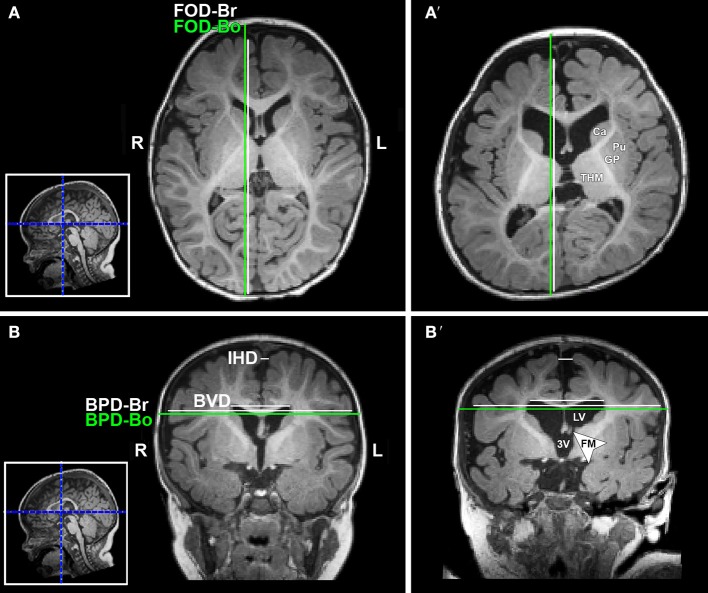
Illustration of linear brain metrics (see Methods) overlaid on T1-weighted axial **(A,A')** and coronal **(B,B')** sections for a full-term naïve control **(A,B)** and full-term patient **(A',B')** that were both scanned at 9 months of age. 3V, third ventricle; BPD, biparietal distance brain (BPD-br) and bone (BPD-bo); BVD, biventricular distance; Ca, caudate; FM, foramen of Monro; FOD, fronto-occipital difference brain (FOD-br) and bone (FOD-bo); GP, globus pallidus; IHD, interhemispheric distance; L, left; LV, lateral ventricle; Pu, putamen; R, right; THM, thalamus.

T1-weighted MRI images were reoriented for uniform head alignment (Figure 2) using Freeview (v.2.0) from Freesurfer (The General Hospital Corporation, Boston, MA) for which 5 landmarks were used: bilateral cochlea (left-right), obex (inferior), posterior commissure (superior/posterior), and anterior commissure (anterior) (34). Two blinded researchers measured 6 linear brain metrics (mm) using ITK-SNAP software (v.3.6.0) (35) as previously published (23–26, 36). An axial section with basal ganglia and thalamus maximally apparent (Figures 3A,A′) was used to measure fronto-occipital diameters of the *brain* (FOD-Br) and *bone* (FOD-Bo) on the right brain hemisphere. A coronal section at the level of the foramen of Monroe (Figures 3B,B′) was used to measure the interhemispheric distance (IHD), biventricular distance (BVD), and biparietal diameters of the *brain* (BPD-Br) and *bone* (BPD-Bo). An additional 4 measures were calculated as follows: (1) **%FOD-difference** = ((FOD-Bo – FOD-Br)/FOD-Bo) x100); (2) **%BPD-difference** = ((BPD-Bo – BPD-Br)/BPD-Bo) x100); (3) **%IHD-to-brain ratio** = ((IHD/BPD-Br) x100); and (4) **%BVD-to-brain ratio** = ((BVD/BPD-Br) x100).

### Volumetric Analysis

T2-weighted image segmentation was performed using Morphologically Adaptive Neonatal Tissue Segmentation (MANTiS) toolbox (27). Although MANTiS was originally designed for application in neonates, we have applied it to infants <12-months-old. Additional FMRIB Software Library (FSL; v.5.0) tools were used for pre-processing and post-segmentation editing (see below). Segmentation involved 4 major steps:

#### Preprocessing

(i) Intracranial space segmentation: T2 images were skull-stripped using the unvalidated “Simple Watershed Scalping” module in the MANTiS toolbox followed by manual editing in FSLview; (ii) Bias field correction using FMRIB's Automated Segmentation Tool (FAST) (37); (iii) Setting image origin using “Origin to the Center of Mass” module in the MANTiS toolbox.

#### MANTiS Segmentation

Preprocessed images underwent MANTiS segmentation pipeline (27). This study focused on the CSF segmentation, comprised of extra-axial space (EAS) and ventricular system. Automated CSF segmentations were visually inspected and subsequently edited to correct for any tissue misclassifications as described below.

#### Post-segmentation Editing

Automated CSF segmentations were (i) masked to zero voxels outside of intracranial space, (ii) thresholded at 40% to eliminate voxels with <40% probability of representing CSF, and (iii) converted to a binary mask. Additional *complex editing* was undertaken due to frequent exclusion of CSF spaces and inclusion of brain tissues. Subsequent partial volume estimate map of CSF generated by FAST (37) was (a) thresholded at 50% to eliminate voxels with <50% of their volume comprising CSF, (b) converted to a binary mask, and subsequently (c) combined with MANTiS' thresholded/binarized CSF mask. In this way, FAST's CSF map filled in CSF spaces missing in MANTiS' CSF segmentation (e.g., cisterns, 4th ventricle, and sulcal spaces). This resulted in a “comprehensive” CSF image, which underwent additional minor manual editing to erase misclassified brain tissue. This final total CSF segmentation was divided into EAS and ventricular system by manually erasing ventricles from CSF segmentation to produce EAS segmentation.

#### Volumetry

**Figure 4:**
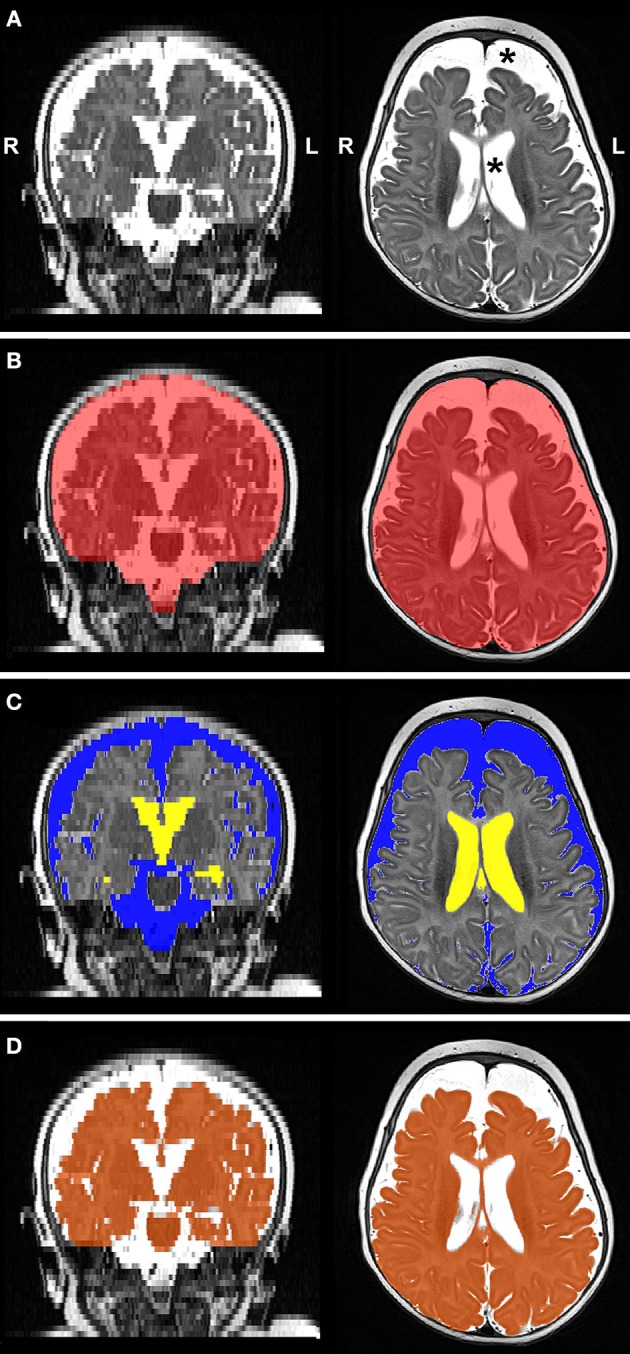
Representative T2-weighted image (**A**; gray-scale) and its final edited MANTiS segmentations of intracranial space (**B**; red), total CSF subdivided into extra-axial space (**C**; blue) and ventricular system (**C**; yellow), and whole brain (**D**; orange) in a full-term patient scanned at 3.4 months of age. Note increased CSF volume (asterisks in Panel A). L, left; R, right.

Figure 4 illustrates representative segmentations used for volumetric analysis. The difference between intracranial and total CSF (for whole brain volume), and total CSF and EAS (for ventricular volume), were calculated. Volumes of each division were reported as absolute volumes (cm^3^) and normalized values as % intracranial volume (%ICV) to correct for interindividual variation (38).

### Statistical Analyses

Statistical analyses were performed using Statistical Package for the Social Sciences (SPSS; v.23.0, IBM Corporation, Armonk, New York). Since inter-observer reliabilities for linear brain metrics were high (>0.8), measures obtained from two blinded researchers were averaged. Normal distribution of continuous variables was confirmed by Shapiro-Wilk test. Absolute measures (mm, cm^3^) were related to group status using a general linear model univariate analysis with corrected age at scan as a covariate. Mean normalized measures (%) were compared between groups using a one-way analysis of variance (ANOVA) with Tukey's honestly significant difference test. Statistical significance was assessed at the α < 0.05.

## Results

### Qualitative Evaluation

**Figure 5:**
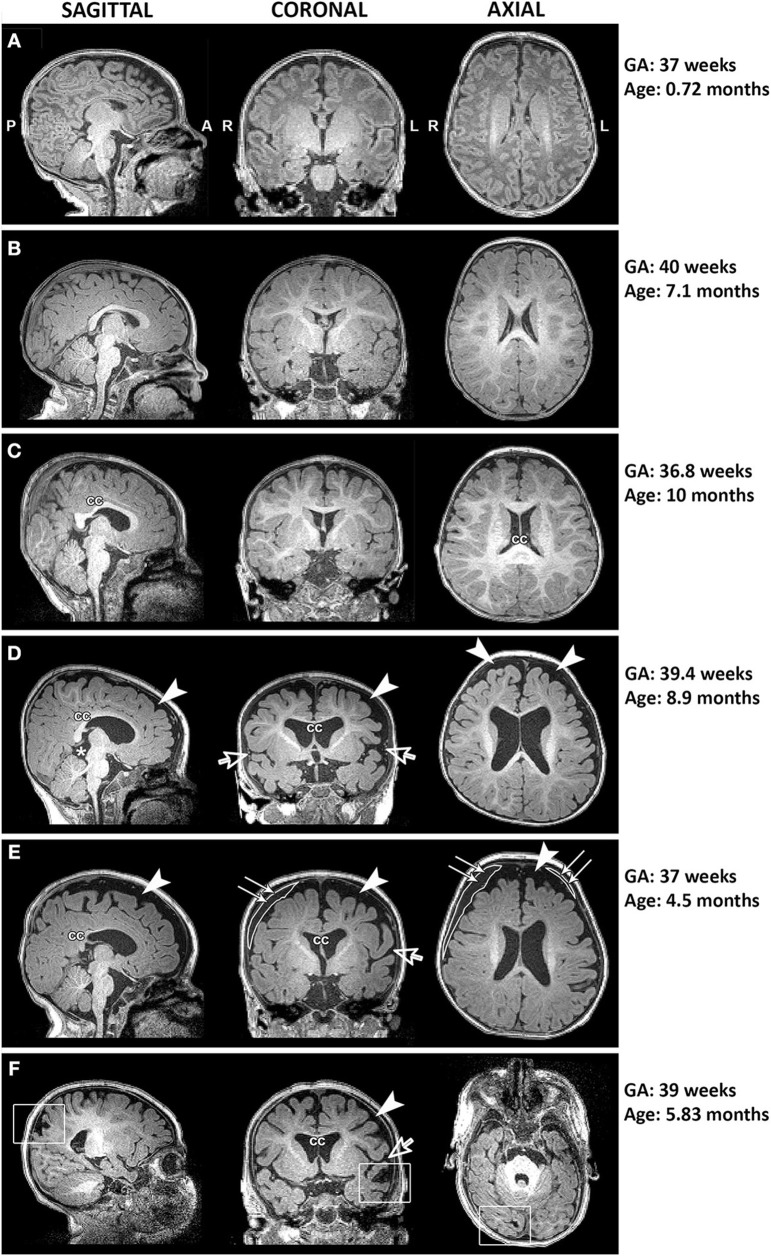
Representative T1-weighted images of full-term infants with typical brain structure **(A,B)** and those with altered corpus callosum (cc; **C–F**), increased extra-axial space (arrowheads) and widened Sylvian fissure (open arrows; **D–F**), presence of cyst (asterisk; **D**), incidental subdural hematoma (double arrows; **E**), and old venous hemorrhagic stroke (square boxes; **F**). L, left; GA, gestational age; R, right.

**Figure 6:**
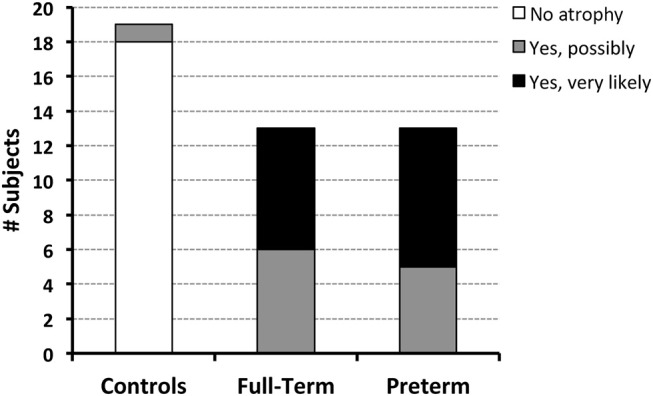
Both full-term and pre-term patients had similar qualitative MRI findings of either *possible* [6/13 (46%) full-term and 5/13 (38%) pre-term patients] or *very likely* brain atrophy [7/13 (54%) full-term and 8/13 (62%) pre-term patients]. In contrast, possible signs of brain atrophy were observed in only 1/19 (5%) full-term naïve controls.

Qualitative evaluation revealed clinically significant incidental MRI findings in both full-term (Figure 5) and premature patients (not shown) without any previously known neurological concerns. Both full-term and premature patients had qualitative MRI findings suggestive of as either *possible* or *very likely* brain atrophy (Figure 6).

### Linear Brain Metrics Results

**Figure 7:**
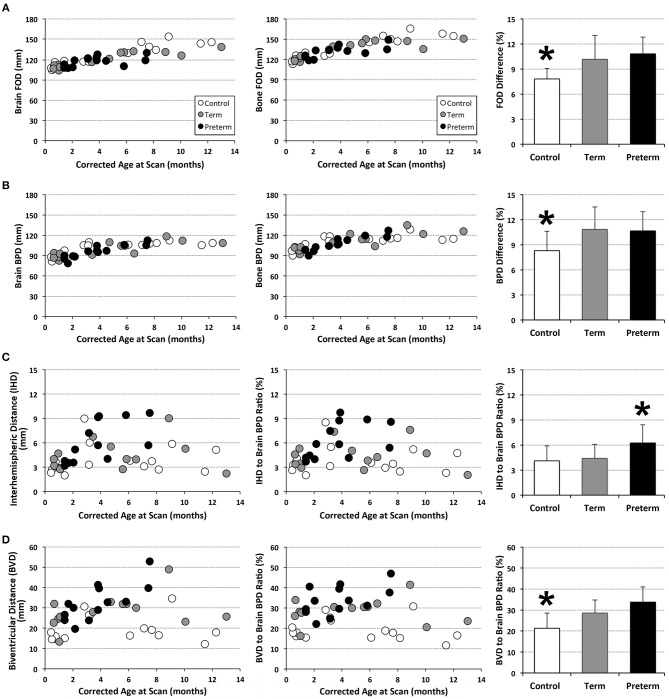
Graphs display individual absolute linear brain measures (mm; **A–D**) and ratio (%) **(C,D)** for full-term (*n* = 13; gray circles) and pre-term patients (*n* = 13; black circles), and full-term naïve controls (*n* = 17; open circles). Bar graphs (±SD) display either mean % difference **(A,B)** or % ratio **(C,D)**. *p < 0.05. FOD, fronto-occipital diameter; BPD, biparietal diameter.

Analysis of T1 images allowed for linear brain metric analysis (Figure 3) in full-term naïve controls (*n* = 17), and full-term and premature patients (*n* = 13/group). The absolute values of both *brain* and *bone* FOD [*brain F*_(1, 39)_ = 115.83, *p* < 0.001; *bone F*_(1, 39)_ = 103.01, *p* < 0.001; Figure 7A) and BPD (*brain F*_(1, 39)_ = 58.85, *p* < 0.001; *bone F*_(1, 39)_ = 50.69, *p* < 0.001; Figure 7B) consistently increased with age, suggesting growth of head and whole brain with age with no differences between groups. However, mean % difference in FOD [*F*_(2, 40)_ = 9.04, *p* < 0.001] and BPD [*F*_(2, 40)_ = 5.28, *p* = 0.09] was significantly lower in controls compared with both full-term and premature patients (*p* < 0.05; Figures 7A,B), suggesting increased EAS in patients. Absolute IHD did not significantly change with age [*F*_(1, 39)_ = 2.15, *p* = 0.15; Figure 7C], but differed between groups [*F*_(2, 39)_ = 3.86, *p* = 0.03]. Mean % IHD-to-*brain* ratio was higher in premature patients [*F*_(2, 40)_ = 5.24, *p* = 0.01] compared to both full-term patients and controls (both *p* < 0.05; Figure 7C) implicating increased interhemispheric space in premature patients only. Finally, while advancing age did not have a significant effect on absolute BVD [*F*_(1, 39)_ = 2.5, *p* = 0.12; Figure 7D], we report group differences [*F*_(2, 39)_ = 7.53, *p* = 0.002] between naive controls and both full-term (*p* < 0.001) and premature (*p* = 0.03) patients (Figure 7D) but not between patient groups. Similarly, mean % BVD-to-*brain* ratio was significantly lower [*F*_(2, 40)_ = 12.18, *p* < 0.001] in naïve controls compared to both full-term (*p* < 0.05) and premature (*p* < 0.01) patients (Figure 7D) implicating increased ventricular size in both patient groups.

### Volumetric Analysis Results

**Figure 8:**
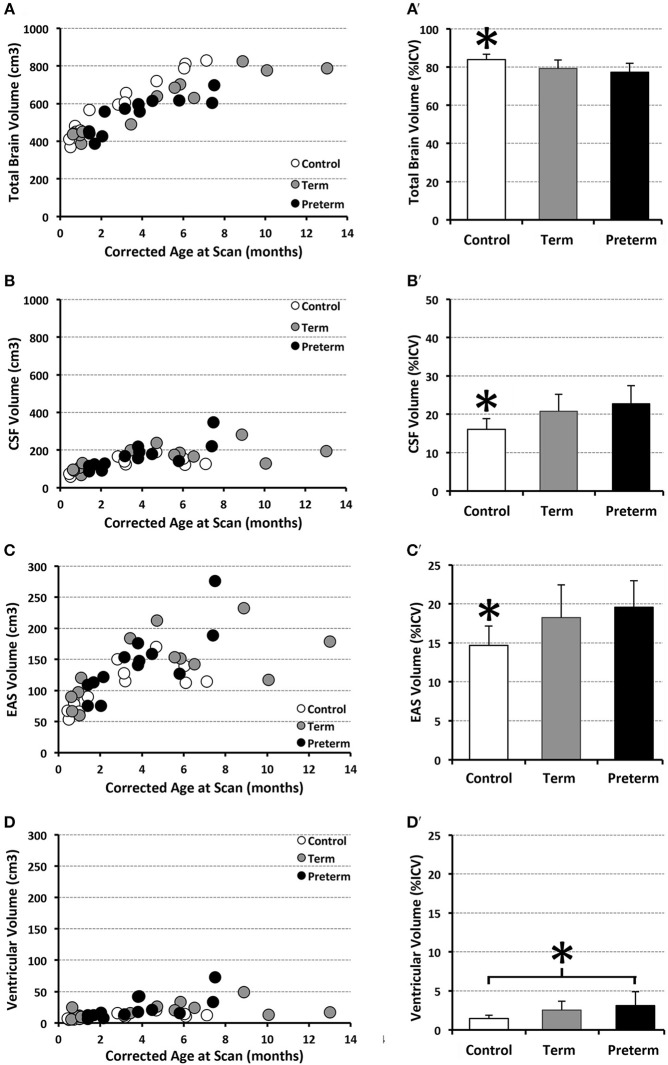
Graphs show individual absolute (scatter plots) and mean normalized volumes (SD; bar graphs) as % intracranial volume (ICV) for whole brain **(A,A')** and total CSF **(B,B')**, as well as CSF sub-divisions of extra-axial space (EAS; **C,C'**) and ventricular volume **(D,D')**. Graphs illustrate 3 groups analyzed: full-term (*n* = 13; gray circles) and pre-term patients (*n* = 13; black circles), and full-term naïve controls (*n* = 13 open circles). *p < 0.05.

Structural segmentation of T2-weighted images (Figure 4) allowed for volumetric analysis (*n* = 13/group) which shows that absolute volumes of the intracranial space [*F*_(1, 35)_ = 131.44, *p* < 0.001; not shown), whole brain [*F*_(1, 35)_ = 159.70, *p* < 0.001; Figure 8A), and total CSF [*F*_(1, 35)_ = 25.07, *p* < 0.001; Figure 8B] increased with age for all groups. An interaction between age at scan and group status was observed for absolute volumes of intracranial space [*F*_(2, 33)_ = 4.10, *p* = 0.03; not shown], whole brain [*F*_(2, 33)_ = 8.47, *p* = 0.001] and CSF [*F*_(2, 33)_ = 3.65, *p* = 0.04], suggesting altered growth trajectories between groups with advancing age. However, group status was independently associated with only absolute whole brain volume [*F*_(2, 35)_ = 8.03, *p* = 0.001; Figure 8A], suggesting brain size is significantly greater in naïve controls compared to both full-term and premature patients (both *p* = 0.001), with no difference detected between patient groups (*p* = 0.94). Mean normative whole brain and, reciprocally, CSF were significantly different [*F*_(2, 36)_ = 9.03, *p* = 0.001; Figures 8A′,B′] between controls and both full-term (*p* < 0.05) and premature (*p* < 0.01) patients. Subsequently, analysis of CSF distribution showed that absolute EAS volumes significantly increased with age [*F*_(1, 35)_ = 27, *p* < 0.001; Figure 8C], in contrast to ventricular volumes which were relatively stable [*F*_(1, 35)_ = 2.12, *p* = 0.16; Figure 8D]. While no group differences were found for absolute EAS volume [*F*_(2, 35)_ = 2.07, *p* = 0.14; Figure 8C], ventricular volumes were significantly greater [*F*_(2, 35)_ = 19.11, *p* < 0.001] in both full-term and premature patients compared to naïve controls (both *p* < 0.001) with no difference between patient groups (*p* = 0.56; Figure 8D). No interaction was observed between age at scan and group status for absolute EAS [*F*_(2, 33)_ = 2.62, *p* = 0.09] or ventricular volumes [*F*_(2, 33)_ = 0.66, *p* = 0.52]. When evaluated as %ICV, mean normative EAS was significantly higher [*F*_(2, 36)_ = 7.09, *p* = 0.003; Figure 8C′] in both full-term (*p* < 0.05) and premature (*p* < 0.01) patients compared to naïve controls, whereas mean normative ventricular volume was significantly higher [*F*_(2, 36)_ = 5.6, *p* = 0.008] in premature patients compared to only controls (*p* < 0.01; Figure 8D′).

## Discussion

To our knowledge, this is the first MRI study showing qualitative and quantitative findings of greater CSF and smaller brain tissue in both full-term and premature infants following life-saving thoracic non-cardiac surgical and complex critical care compared to controls. These results suggest similar risk for brain injury and brain atrophy for full-term and premature infants in the context of complex perioperative care for the LGEA. Despite observed differences in global brain size in this study, total intracranial volume was not significantly different among the groups suggesting head circumference may not be a reliable index of brain growth in selected group of infants born with LGEA.

### Qualitative and Quantitative Brain Findings

Consistent with our qualitative findings, a recent study by Stolwijk et al. (16) reported a high incidence of brain injury (viz. non-parenchymal abnormalities, including intraventricular and subdural hemorrhages) in patients following neonatal surgery for major non-cardiac congenital anomalies (16, 18). With respect to whole brain volumetry, one study found similar brain volumes in premature infants free of any significant medical problems at term-equivalent age compared to full-term infants (39). In contrast, there is an abundance of evidence suggesting prematurity is associated with both risk of brain injury and atrophy [see reviews (7, 40)]. Lower gestational age at birth has been associated with smaller brain volumes, as estimated by head ultrasound (41) and MRI (42–44).

### Cerebrospinal Fluid

Our study also adds knowledge about higher CSF volumes in a unique cohort of full-term and moderate-to-late premature infants following complex thoracic non-cardiac perioperative critical care for LGEA. Similar findings were observed in several large cohort studies, reporting significantly greater CSF in pre-mature infants compared to full-term infants at term-equivalent age (42–45). In the present study, enlarged EAS was observed in both full-term and pre-term patients compared to healthy controls, whereas ventricles were only significantly enlarged in pre-term patients relative to controls. Benign extra-axial fluid enlargement (viz. idiopathic external hydrocephalus without evidence of ventricular enlargement or hydrocephalus) has been extensively reported in the literature (46–50) since the early 1980s and has been associated with both prematurity (47, 49) and previous ECMO exposure (51–53).

### Significance for Neurodevelopmental Outcomes

Future follow-up studies should determine the neurodevelopmental outcomes of full-term and premature infants born with LGEA in relation to their estimated brain and CSF volumes.

#### Brain Volume Decrease

While efforts to elucidate typical brain development in full-term infants using neuroimaging have emerged (54–56), the significance of whole brain volume during the first year of life in relation to long-term outcome remains poorly understood. One study in moderate-to-late pre-term infants reported an association between larger brain volumes at term-equivalent age and higher cognitive and language scores at 2 years of age (57). However, another study in full-term infants with neonatal encephalopathy showed a significant association between brain volumes at 6 months old and language scores, but not motor or cognitive scores (58).

#### CSF Increase

Preterm infants with significantly greater CSF at term-equivalent age are at increased risk of moderate-to-severe disability at 1 year of age (45) and cognitive and language scores at 2 years of age (57). Another study of pre-term infants (*n* = 12) by Keunen et al. (59) showed that ventricular volume at term-equivalent age was inversely related to all measures of neurodevelopment, which persisted through early school age. Furthermore, the presence of greater extra-axial fluid has been associated with motor and neurodevelopmental delays in premature infants (60–63). A similar study in full-term infants (64) reported greater EAS in the absence of ventricular dilatation that may represent an early brain MRI phenotype of autism spectrum disorder (64). Specifically, the authors reported significantly greater extra-axial fluid by 6–9 months of age that persisted into the second year of life in those infants later diagnosed with autism.

#### Underlying Disease

Children with congenital gastrointestinal anomalies experience multiple stressors while hospitalized early in life (65). Early stress and inadequate nutrition in infancy are linked to altered growth patterns (66, 67) and later neurodevelopmental delays (68, 69). It was shown that children with congenital gastrointestinal anomalies have similar growth and body composition to their peers (70). However, like pre-term-born children (71, 72), higher fat-free mass (but not fat mass) later in life is associated with higher cognitive test scores in children with congenital gastrointestinal anomalies (70). Authors in the latter study concluded that closer tracking of body composition and interventions aimed at increasing fat-free mass may improve long-term outcomes in this population.

#### Neonatal Surgery

It was previously reported that neonatal surgery for major birth defects was associated with neurodevelopmental delay at 2 years of age, suggesting long-term adverse sequelae in the setting of critical illness and surgery (17, 18, 73).

### Study Limitations

Findings reported in the present study must be interpreted in the context of several limitations. As a pilot study, a small sample size, slight incongruences in age range between groups, and diagnoses within patient groups are potential limitations. This study lacked a true control group due to the absence of infants with similar non-cardiac LGEA that undergo alternative treatment. We were unable to recruit infants that received only prolonged sedation. Our recent study (74) showed that a very small number of full-term infants that were admitted for treatment of pneumonia were usually treated <5 days - before the onset of physical dependence to sedation. Although motion during non-sedated MRI scan acquisition remains a significant challenge (75, 76) our efforts to refine scan protocols allowed for improved rates of successful scan completion (100% of scanned patients). Furthermore, gender differences have been previously reported for brain tissue (43, 77, 78) and lateral ventricular volumes (64). In this study, MRI scans for volumetric quantification were dominated by male control infants, whereas sex distributions in both patient groups were relatively even between the two sexes. Understanding possible sex differences should be a subject of future studies. In light of aforementioned limitations, it is not feasible to say whether reported findings are due to (i) unknown underlying biology/genetics, (ii) critical illness, (iii) aspects of associated critical care treatment (e.g., neonatal surgery, cumulative anesthesia, and prolonged sedation exposure), (iv) altered feeding (viz. nitrogen balance in the setting of parenteral nutrition), and/or (v) social deprivation. Future studies are needed to establish the relationship between reported incidental MRI findings, brain volumes and long-term neurodevelopmental outcomes, as well as elucidate whether full-term infants from our cohort have differential plasticity/adaptations in overcoming such insults during the critical early period of brain development.

## Author Contributions

Authorship credit was based on substantial contributions to (1) the concept and manuscript design [CM, DB], (2) acquisition and analysis of data, or interpretation of data [all authors], (3) drafting the article or revising it critically for important intellectual content [all authors], (4) final approval of the version to be published [all authors], and (5) accountability for all aspects of the work in ensuring that questions related to the accuracy or integrity of any part of the work are appropriately investigated and resolved [all authors].

### Conflict of Interest Statement

The authors declare that the research was conducted in the absence of any commercial or financial relationships that could be construed as a potential conflict of interest.

## Abbreviations

ANOVA: analysis of variance
BPD-Bo, biparietal diameter: bone
BPD-Br, biparietal diameter: brain
BVD: biventricular distance
CSF: cerebrospinal fluid
EAS: extra-axial space
ECMO: extracorporeal membrane oxygenation
FAST: FMRIB's Automated Segmentation Tool
FSE: Fast Spin Echo
FOD-Bo, fronto-occipital diameter: bone
FOD-Br, fronto-occipital diameter: brain
FOV: field of view
FSL: FMRIB Software Library
GA: gestational age
ICC: intraclass correlation coefficient
ICV: intracranial volume
IHD: interhemispheric distance
LGEA: long-gap esophageal atresia
MANTiS: morphologically adaptive neonatal tissue segmentation
MEMPRAGE: Multi-Echo Magnetization Prepared Rapid Acquisition Gradient Echo
MRI: magnetic resonance imaging
PICC: peripherally inserted central catheter
SPSS: Statistical Package for the Social Sciences
TE: echo time
TR: repetition time.
